# Clinical and Immunological Factors Associated with Recommended Trough Levels of Adalimumab and Infliximab in Patients with Crohn’s Disease

**DOI:** 10.3389/fphar.2021.795272

**Published:** 2022-01-03

**Authors:** Beatriz Orts, Ana Gutierrez, Lucía Madero, Laura Sempere, Ruben Frances, Pedro Zapater

**Affiliations:** ^1^ Unidad de Farmacología Clínica, Hospital General Universitario de Alicante, Alicante, Spain; ^2^ Instituto de Investigación Sanitaria y Biomédica de Alicante (ISABIAL), Alicante, Spain; ^3^ Servicio de Medicina Digestiva, Hospital General Universitario de Alicante, Alicante, Spain; ^4^ CIBERehd, Instituto de Salud Carlos III, Madrid, Spain; ^5^ Clinical Medicine Department, Universidad Miguel Hernández, Elche, Spain; ^6^ Instituto de Investigación, Desarrollo e Innovación en Biotecnologia Sanitaria de Elche, IDiBE, Universidad Miguel Hernández, Elche, Spain

**Keywords:** crohn’s disease, anti-TNF, cytokines, pharmacokinetics, trough serum levels

## Abstract

**Introduction:** Up to 40% of patients with Crohn’s disease do not respond to treatment with anti-TNF or lose response after the initial benefit. Low drug concentrations have been proposed as the main predictor of treatment failure. Our aim was to study the immunological profile and clinical evolution of patients with Crohn’s disease according to the anti-TNF dose and serum trough levels.

**Methods:** Crohn’s disease patients in remission treated with infliximab or adalimumab at stable doses for at least for 3 months were included. Serum levels of anti-TNF, TNF-α, interferon-γ, and interleukin IL-12, IL-10, and IL-26 were determined in blood samples taken just before drug administration. Patients were classified according to anti-TNF levels below, within, or above the target level range and the use of intensified doses. Clinical evolution at 6 months was analyzed.

**Results:** A total of 62 patients treated with infliximab (8 on intensified schedule) and 49 treated with adalimumab (7 on intensified schedule) were included. All infliximab-treated patients showed levels within the recommended range, but half of adalimumab-treated patients were below the recommended range. A significant negative relationship between body weight and adalimumab levels was observed, especially in patients treated with intensified doses. Patients with infliximab levels over 8 µg/ml presented higher median IL-10 than patients with in-range levels (84.0 pg/ml, interquartile range [IQR] 77.0–84.8 vs. 26.2 pg/mL, IQR 22.6–38.0; p < 0.001), along with lower values of interferon-γ (312.9 pg/ml, IQR 282.7–350.4 vs. 405.6 pg/ml, IQR 352.2–526.6; p = 0.005). Patients receiving intensified versus non-intensified doses of infliximab showed significantly higher IL-26 levels (91.8 pg/ml, IQR 75.6–109.5 vs. 20.5 pg/ml, IQR 16.2–32.2; p = 0.012), irrespective of serum drug levels. Patients with in-range levels of adalimumab showed higher values of IL-10 than patients with below-range levels (43.3 pg/ml, IQR 35.3–54.0 vs. 26.3 pg/ml, IQR 21.6–33.2; *p* = 0.001). Patients treated with intensified vs regular doses of adalimumab had increased levels of IL-12 (612.3 pg/ml, IQR 570.2–1353.7 vs. 516.4 pg/mL, IQR 474.5–591.2; *p* = 0.023). Four patients with low adalimumab levels (19%) and four treated with intensified doses were admitted to a hospital during a follow-up compared to none of the patients with levels within the range.

**Conclusion:** Patients with Crohn’s disease treated with infliximab and adalimumab exhibit differences in serum levels of cytokines depending on the drug, dose intensification, and steady state trough serum levels.

## Introduction

Crohn’s disease (CD) is an immunologically mediated inflammatory disease of the gastrointestinal tract. Biologic drugs, especially antitumor necrosis factor-alpha (anti-TNF), are widely used in the induction and maintenance of disease remission. Two mechanisms of action of anti-TNF have been proposed: the induction of T-cell apoptosis and the Fc-receptor–dependent promotion of reparative wound-healing macrophages to explain both anti-inflammatory and promotion of mucosal healing effects showed by these drugs (Levin et al., 2016).

In experimental models of colitis, pathogenic T-cell responses are driven by IL-12 and IL-23 production by mononuclear phagocytes and deficits in IL-10 and TGF-β pathways (Arnold et al., 2016), suggesting first, the involvement of the cytokine network in either promoting or suppressing inflammation (Friedrich et al., 2019) and, second, a possible interference by the anti-TNF mechanism of action. Infliximab therapy in patients with Crohn’s disease has been associated with reduced lymphocyte populations in intestinal mucosa and a lower ability of these cells to produce IL6 and IL10 “*in vitro*” (Cornillie et al., 2001).

Infliximab and adalimumab are the main anti-TNF drugs used in the treatment of CD. Numerous observational studies have associated a higher risk of loss of clinical response with the presence of antibodies against adalimumab or infliximab whereas high trough levels of drugs have been associated with greater clinical response rates (Kennedy et al., 2019).

Although, the use of anti-TNF has significantly improved the results of CD treatment, up to 40% of patients do not respond or lose response after the initial benefit (Katsanos et al., 2019). Low drug concentrations, in part by the presence of antibodies against the drug, have been described as the main predictor of anti-TNF treatment failure in anti-TNF–naive patients with active luminal CD (Kennedy et al., 2019). Antibodies against the drug are thought to promote drug clearance, and they correlate with higher markers of inflammation such as C-reactive protein (CRP) (Nguyen et al., 2015). Previously, our group has described the relationship between serum levels of anti-TNF and IL-10 in patients without anti-drug antibodies, suggesting a link between the systemic immune activation status and anti-TNF concentrations in CD patients (Zapater et al., 2019). Previous studies have not considered whether anti-TNF serum trough levels are in the recommended doses when investigating their interactions with the inflammatory response in this setting. This is in part due to collection of single blood samples, precluding estimation of the pharmacokinetic parameters of the drug in individual patients. The aim of this study was to identify anti-TNF–treated CD patients, without anti-drug antibodies and unexpected anti-TNF serum trough levels, comparing their immunological profiles and clinical evolution in patients with expected anti-TNF pharmacokinetics.

## Materials and Methods

Consecutive CD patients diagnosed and controlled in the area of Alicante, Spain, were included in this prospective observational study. The diagnosis of CD was established according to standard clinical, endoscopic, histological, and radiographic criteria. Patients were included if they were treated for at least three months with stable doses of infliximab (5 mg/kg 8-weekly) or adalimumab (40 mg every other week); or if they received anti-TNF intensified therapy, defined as either an increased dose or shortened dosing interval (infliximab 5 mg/kg 6-weekly or 10 mg/kg 8-weekly, adalimumab 40 mg each week or every 10 days). Patients treated with antibiotics in the previous 4 weeks, those with signs of active infection, and those who refused to sign informed consent to participate in the study were excluded. The Ethics Committee from Hospital General Universitario de Alicante approved the study protocol.

The usual clinical and analytical variables in the management of CD patients, including fecal calprotectin, were recorded in all patients at baseline and at 6 months of follow-up. The number of disease flares and the need for hospitalization or surgery during the 6-month follow-up period were also collected.

At inclusion, blood samples taken just before anti-TNF administrations were used for hematological, biochemical, and immunological studies and for infliximab and adalimumab trough serum level determination.

### Estimation of Pharmacokinetic Parameter

Pharmacokinetics models for infliximab and adalimumab were built using published population pharmacokinetic parameters (clearance, volume of distribution, intercompartmental clearances, and absorption rate constant) (Fasanmade et al., 2011; Ternant et al., 2015; Berends et al., 2018) and from the official information on drugs available on the websites of regulatory agencies (EMA and FDA). Simulations of ordinary differential equation models were built using R software RxODE (Wang et al., 2016). Specifically, these models were used to simulate populations of 10,000 patients treated with standard doses of adalimumab or infliximab to obtain the trough levels of the drug that would be reached in a steady-state situation. These populations were used as references to identify the percentile corresponding to the trough levels measured in our patients.

Target steady-state trough levels appropriate to achieving clinical remission in luminal IBD were 3–8 μg/ml for infliximab and 5–12 μg/ml for adalimumab according to published consensus (Mitrev et al., 2017; Papamichael et al., 2019).

### Detection of Serum Cytokine and Free Anti-TNF-α Levels

The presence of anti-drug antibodies and serum levels of TNF-α, interferon-γ, and interleukin (IL)-12p40, IL-10, and IL-26 were determined by enzyme-linked immunosorbent assays (ELISAs) using Human Quantikine kits from R&D Systems (Minneapolis, MN, United States). ELISAs were also carried out to measure free infliximab and adalimumab levels and to detect anti-drug antibodies (Matriks Biotek, Ankara, Turkey), according to the manufacturer’s instructions. All samples were tested in triplicates and read in a Sunrise microplate reader (Tecan, Männedorf, Switzerland). The detection limit for each cytokine assay varied between 2 and 5 pg/ml and between 0.1 and 0.3 μg/ml in the case of free anti–TNF-α kits. Standard curves were generated for every plate, and the average z standard optical densities were subtracted from the rest of the standards and samples to obtain a corrected concentration for all parameters. The presence of anti-drug antibodies was evaluated by a cutoff value estimated by multiplying the optical density (OD) of the zero standard by 3, as indicated by the manufacturers. Samples were considered positive when the ratio sample OD/0 standard OD was higher than 3.

### Statistical Analysis

Descriptive statistics were expressed as means and standard deviations for continuous variables following a normal distribution or medians and interquartile ranges (IQRs) for noncontinuous variables. Categorical variables were described by frequencies and percentages. Percentile values for theoretical steady state trough levels of anti-TNF (calculated from pharmacokinetics models of infliximab and adalimumab built using population pharmacokinetic parameters) were calculated and categorized as low (<P25), low-medium (P25–P50), medium (P50–P75), and high levels (>P75), and the steady state trough levels measured in patients were classified into one of these categories.

Comparisons between groups were carried out using the chi-square test for categorical variables and the t test or the Mann–Whitney U test for quantitative variables, depending on the normality of the distribution of data. Normality was evaluated with the Shapiro–Wilk test. A univariable linear regression analysis was conducted to assess the association of clinical and experimental variables with trough drug levels. Variables achieving statistical significance (*p* < 0.05) were considered in a multivariable linear regression model. The fit of the linear regression models was determined by the coefficient of determination (R2). The Kolmogorov–Smirnov test, probability–probability plot, and the scatter plot of residuals vs predicted values were performed to check that parametric assumptions of the linear regression model could be assumed.

All tests for significance were conducted using a 2-sided approach with a 5% significance level. The Bonferroni correction was performed for multiple comparisons. All statistical analyses were performed using SPSS software (IBM Corp. Released 2020. IBM SPSS Statistics for Windows, Version 27.0. Armonk, NY: IBM Corp).

## Results

A total of 62 patients treated with infliximab and 49 treated with adalimumab were included. Clinical and demographic characteristics of patients between groups were similar (Table 1). Previous surgery was more frequent in patients on adalimumab whereas concomitant use of azathioprine was more frequent in patients on infliximab.

**TABLE 1:** Characteristics of Crohn’s disease patients included in the study.

Dosing	Infliximab-treated		Adalimumab-treated	
	5 mg/kg 8-weekly	Intensified dose	40 mg every other week	Intensified dose
N	54	8	42	7
Age (yr.)	38 ± 14	38 ± 15	40 ± 12	40 ± 14
Female gender (%)	28 (51.9)	4 (50.0)	21 (50.0)	4 (57.1)
Illness duration (months)	105 [48, 156]	73 [45, 128]	120 [60, 228]	56 [47, 77]
Current smokers yes (%)	19 (39.6)	4 (57.1)	15 (35.7)	2 (28.6)
Weight (Kg)	72.6 ± 18.3	72.8 ± 25.5	71.6 ± 19.8	69.7 ± 15.7
CRP (mg/dl)	0.3 [0.1, 1.0]	1.3 [0.2, 2.1]	0.3 [0.2, 1.4]	0.2 [0.1, 0.4]
CDAI	90 [64, 168]	144 [80, 305]	88 [50, 151]	63 [50, 127]
Previous surgery (%)	10 (20.4)	3 (37.5)	18 (42.9) *	0 (0.0)
Calprotectin (µg/g)	65 [50.0, 215.0]	88 [80.0, 120.0]	65 [39.5, 182.5]	55 [37.3, 75.3]
Albumin (g/dl)	4.0 [3.7, 4.4]	3.8 [2.9, 4.0]	4.1 [3.6, 4.4]	4.3 [3.5, 4.5]
Hematocrit (%)	42.0 [39.8, 43.9]	40.6 [37.4, 43.9]	40.9 [38.6, 43.5]	43.6 [38.2, 45.6]
Leucocytes (/mm^3^)	6840 [5360, 8810]	6853 [6232, 7655]	8100 [5990, 10000]	7300 [5115, 9120]
Montreal (age of onset), No. (%)				
A1	6 (11.0)	1 (12.5)	2 (4.8)	0 (0.0)
A2	40 (74.0)	5 (62.5)	37 (88.1)	6 (85.7)
A3	8 (15.0)	2 (25.0)	3 (7.1)	1 (14.3)
Montreal (location), No. (%)				
L1	15 (27.7)	3 (37.5)	15 (35.7)	1 (14.3)
L2	15 (27.7)	4 (50.0)	7 (16.7)	1 (14.3)
L3	21 (39.0)	0 (0.0)	18 (42.9)	4 (57.1)
L4	3 (5.6)	1 (12.5)	2 (4.8)	1 (14.3)
Montreal (behavior), No. (%)				
B1	35 (64.8)	6 (75.0)	23 (54.8)	2 (28.6)
B2	7 (13.0)	1 (12.5)	6 (14.3)	2 (28.6)
B3	12 (22.2)	1 (12.5)	13 (31.0)	3 (42.9)
Concomitant treatments				
NSAID, No. (%)	2 (3.7)	1 (12.5)	1 (2.4)	0 (0.0)
Azathioprine, No. (%)	22 (40.7)	1 (12.5)	5 (11.9)^a^	2 (28.6)
Steroids, No. (%)	6 (11.1)	2 (25.0)	8 (19.0)	1 (14.3)

Numerical variables are shown as mean ± standard deviation or medians (interquartile range). Categorical variables are described as frequency (percentage). CRP = C-reactive protein.

a *p*<0.05 non-intensified infliximab *vs*. non-intensified adalimumab.

**FIGURE 1:**
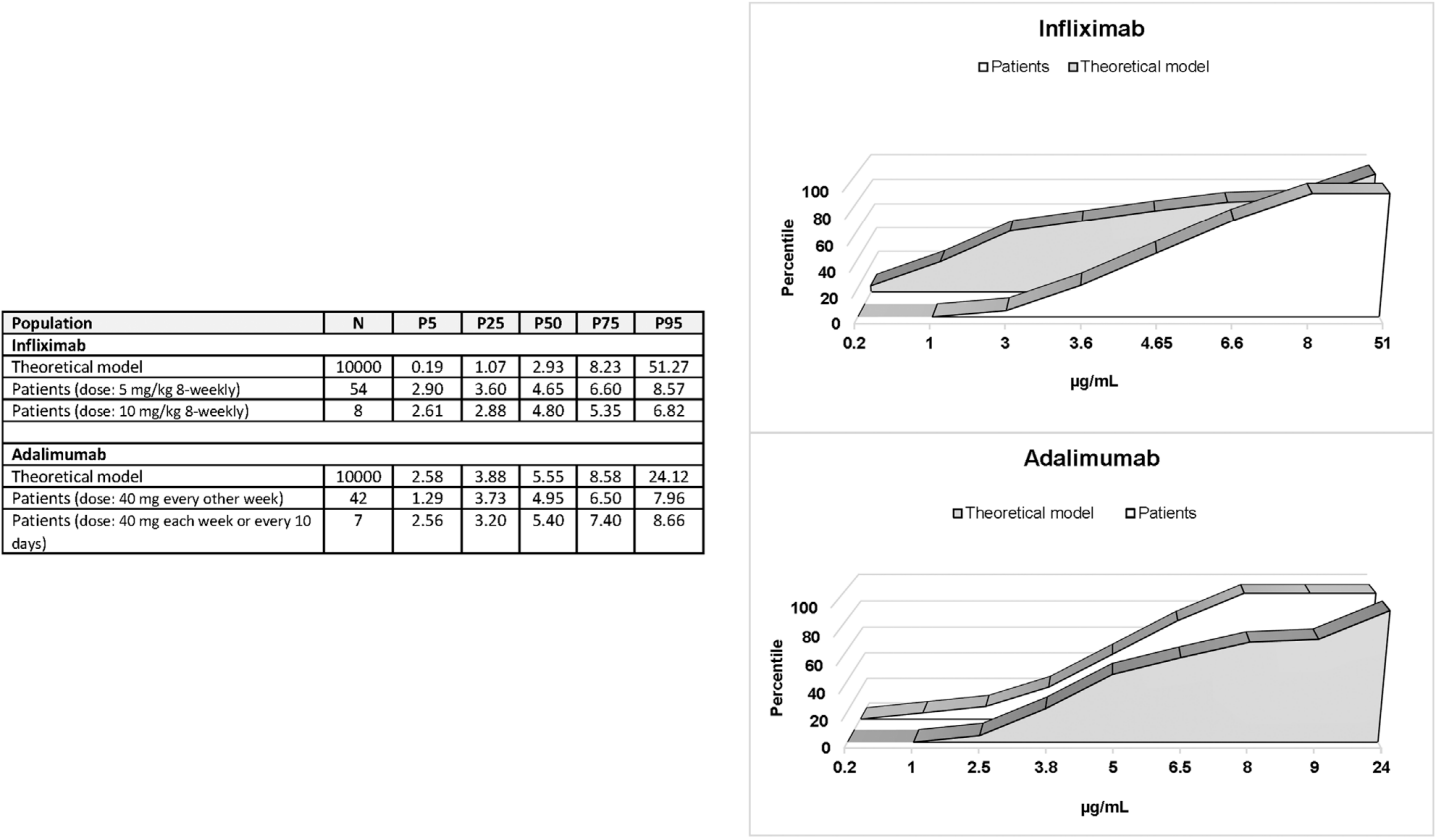
Percentiles 5, 25, 50, 75, and 95 of steady-state trough levels of infliximab and adalimumab in patients compared with the percentile distribution of expected levels.

Patients treated with non-intensified infliximab showed higher drug levels than expected according to published pharmacokinetic parameters while patients treated with non-intensified adalimumab showed serum drug levels similar to those predicted (Figure 1). The built models of infliximab and adalimumab pharmacokinetics are summarized in Supplementary Figures S1, S2. Practically, all patients treated with infliximab showed steady-state trough levels considered as appropriate to achieve clinical remission in luminal IBD according to clinical consensus (3–8 μg/ml). However, half the patients treated with adalimumab were below the recommended range (5–12 μg/ml), although this condition was expected based on the pharmacokinetic parameters of the drug (Figure 1). Eight and seven patients were on intensified infliximab or adalimumab schedules, respectively. The distribution of trough values was similar to that observed in patients on non-intensified schedules (Figure 1).

### Characteristics of Patients Treated With Infliximab According to Categories of Steady-State Trough Levels of Anti-TNF

All patients treated with non-intensified infliximab were distributed in medium (P50-P75) and high (>P75) level categories predicted by pharmacokinetic parameters of the drug. Predicted medium levels (2.93–8.23 μg/ml) exactly match the recommended range of infliximab levels according to clinical consensus (3–8 μg/ml). Eight patients had levels above the recommended range (>8 μg/ml). These patients with high concentrations of infliximab showed similar characteristics to patients with in-range levels except for gender distribution as all patients with high levels were male (Supplementary Table S1). Similarly, patients treated with intensified doses of infliximab showed the same characteristics as patients with levels in the recommended range (Supplementary Table S1). None of the clinical and demographic variables or dosing showed a significant association with infliximab concentrations in the linear regression analysis.

### Values of Cytokine Concentrations Observed in Patients Treated With Infliximab According to Categories of Steady-State Trough Levels of Anti-TNF

**FIGURE 2:**
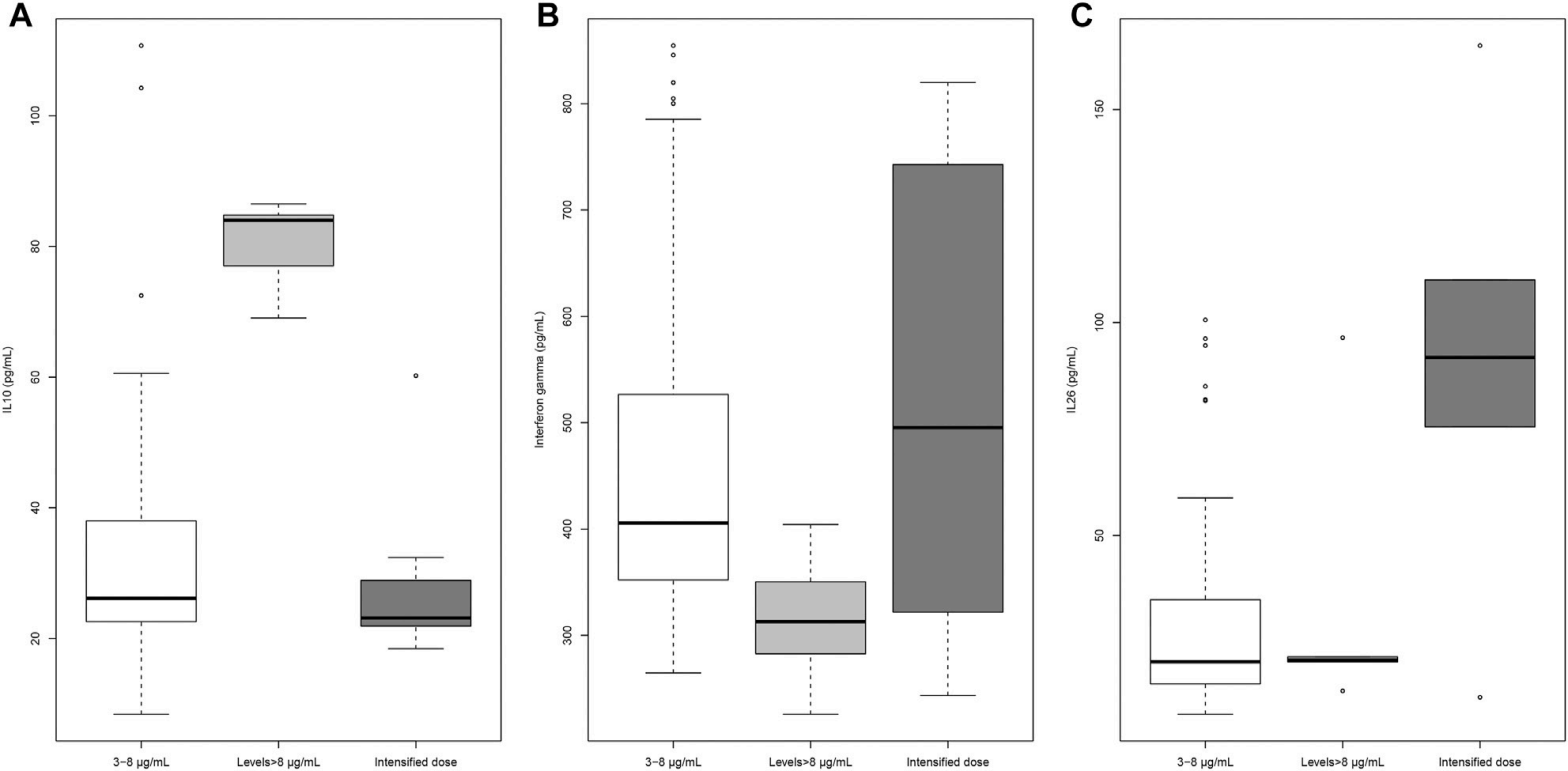
Cytokine profile of patients treated with infliximab.

Infliximab levels were significantly associated with TNF-α (*β* = −0.023; *p* = 0.0078), IL-10 (*β* = 0.044; *p* = 0.00001), IL-12 (*β* = −0.0025; *p* = 0.0018), and interferon-γ (*β* = −0.0049; *p* = 0.0003) in univariable linear regression analyses. Only IL-10 maintained the significance in multivariate analysis (*β* = 0.033; *p* = 0.0028). Patients with trough levels of infliximab higher than 8 μg/ml had either increased values of IL-10 or decreased values of interferon-γ compared with patients with levels in the recommended range (Figure 2). IL-26 levels were significantly higher in patients on intensified vs non-intensified schedule, irrespective of infliximab trough levels (Figure 2). TNF-α and IL-12 showed no significant differences between the three groups (Supplementary Table S2).

### Results on Infliximab-Treated Patients at a 6-month Follow-Up

Of the 47 patients (19.1%), nine patients with infliximab levels in the recommended range showed an active disease (CDAI >150) at 6 months, a similar percentage to that in patients above the range (1 out of 7 patients; 14.3%; *p* = 0.9318) and in those treated with intensified doses (3 out of 8 patients; 37.5%; *p* = 0.4847). Three patients in the recommended range were admitted to hospital during the follow-up, compared to none above the range and 3 in the intensified-dose group. No patient underwent surgery.

### Characteristics of Patients Treated With Adalimumab According to Categories of Steady-State Trough Levels of Anti-TNF

**FIGURE 3:**
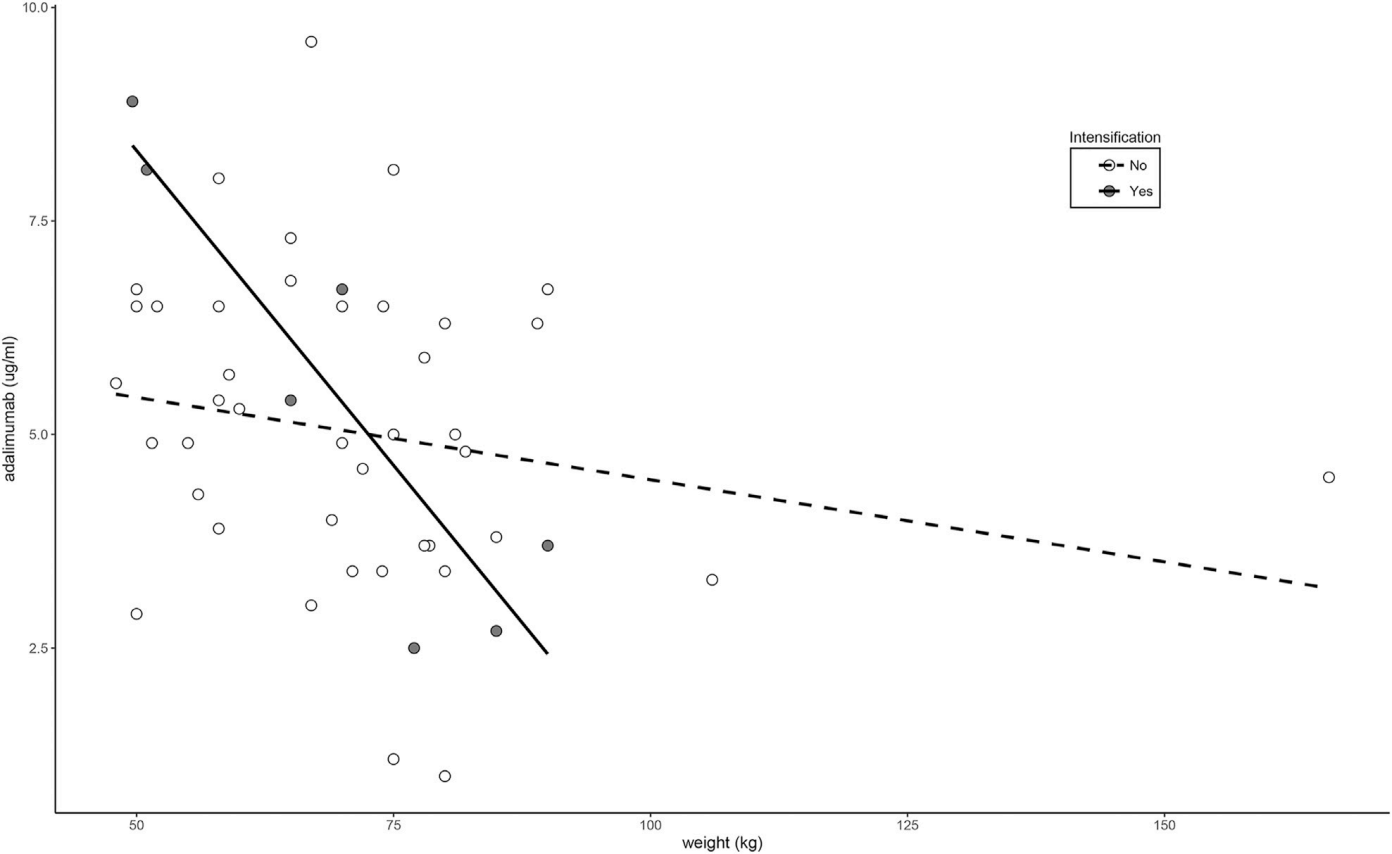
Relationship between weight and adalimumab levels according to dose intensification regimen.

Fifty percent of patients treated with non-intensified adalimumab showed serum concentrations below the recommended range in clinical consensus (<5 μg/ml). These patients showed similar characteristics to those in patients with in-range levels (5–12 μg/ml) (Supplementary Table S3) and with patients treated with intensified doses (Supplementary Table S3). Only the body weight showed a significant association with adalimumab concentrations in the linear regression analysis (*β* = -0.0303; *p* = 0.0397). This association was highly significant in patients treated with intensified doses of adalimumab (*β* = −0.1474; *p* = 0.0062) but weak and non-significant in patients treated with non-intensified doses (*β* = −0.0193; *p* = 0.1973) as shown in Figure 3.

### Values of Cytokine Concentrations Observed in Patients Treated With Adalimumab According to Categories of Steady-State Trough Levels of Anti-TNF

**FIGURE 4:**
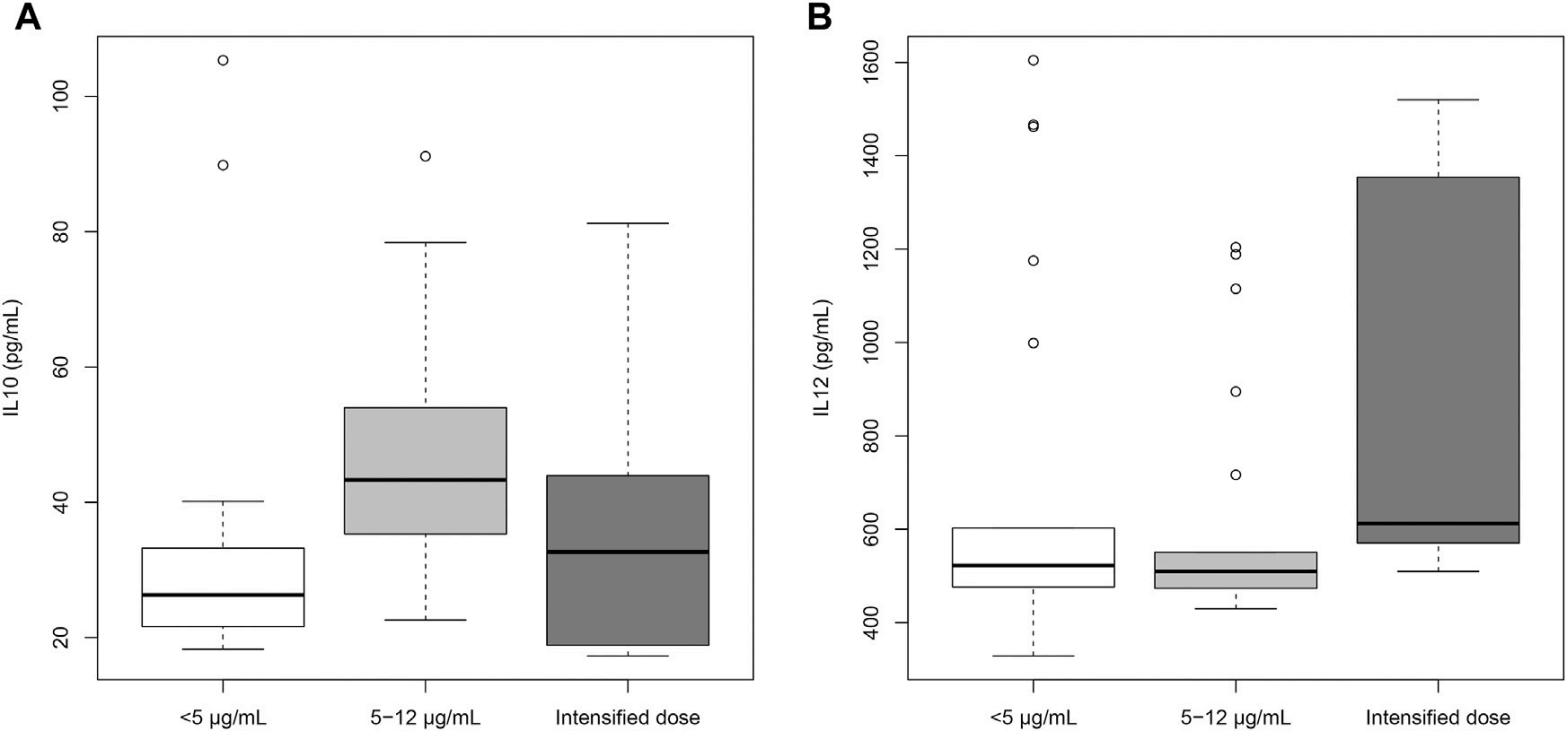
Cytokines profile of patients treated with adalimumab.

Adalimumab levels were significantly associated with IL-10 (β = 0.031; *p* = 0.0173) and IL-12 (β = −0.0018; *p* = 0.0252) in the univariable linear regression analysis. These associations were weak, and the two variables lost their significance in the multivariate analysis. Patients with recommended trough levels of adalimumab showed higher values of IL-10 than patients below the recommended range (Figure 4). Patients treated with intensified doses of adalimumab had increased levels of IL-12 (Figure 4). There were no significant differences in the levels of TNF-α and IL-26 between the three groups (Supplementary Table S4).

### Results in Adalimumab-Treated Patients at a 6-month follow-up

Seven of the 21 patients with adalimumab levels of less than 5 μg/ml (33%) and 8 of the 21 patients with levels in the recommended range (38%) showed an active disease (CDAI >150) at 6 months. The presence of an active disease was observed in four patients treated with intensified doses of adalimumab (57%). Four patients with low adalimumab levels (19%) were admitted to a hospital during the follow-up compared with none of the patients with levels of 5 and 12 μg/ml (0%; *p* = 0.115). The 57% of patients treated with intensified doses were admitted to hospital (4 out of 7 patients; *p* = 0.002 versus patients with levels into the recommended range). One patient in each group underwent surgery.

## Discussion

Anti-TNF therapy has been shown to be effective in CD patients in clinical trials, but only one-third of patients remain in clinical remission at 1 year. A possible explanation for this loss of response is the high intra- and inter-variability of pharmacokinetics of anti-TNF influenced by factors such as dose, route of administration, serum proteins, gender, body weight, systemic inflammation, and the development of immunogenicity (Nguyen et al., 2015). Consequently, numerous studies have been carried out to determine anti-TNF levels that can serve as a guide to identify patients who may become non-responders or may lose response over time. The result of these studies has been translated into ranges of the trough concentrations of anti-TNF (3–8 μg/mL for infliximab and 5–12 μg/mL for adalimumab) associated with a greater probability of maintaining remission. These consensus ranges are based on the results of numerous studies, many of them retrospective (Mitrev et al., 2017; Papamichael et al., 2019), and their amplitude reflects a great variability.

We prospectively analyzed the serum concentrations of anti-TNF in trough samples at a steady state of 62 patients treated with infliximab and 49 treated with adalimumab. Only patients without the presence of anti-TNF antibodies were included in the study to avoid the main known factor associated with alterations in the values of anti-TNF trough concentrations. Initially, we modeled, using known pharmacokinetic parameters, the steady-state trough concentrations of infliximab and adalimumab that would be expected in a population of 10,000 patients with CD.

In the case of infliximab, 95% of the patients in our study had concentrations above the 50th percentile of the expected concentrations according to the published pharmacokinetic parameters. Several factors associated with an increased clearance of the drug and lower concentrations have been identified in population pharmacokinetic studies of infliximab, such as body weight, the presence of anti-infliximab antibodies, albumin concentrations (Matsuoka et al., 2020; Hanzel et al., 2021), the concomitant use of immunosuppressants, and the degree of systemic inflammation (Hemperly and Vande Casteele, 2018). We cannot rule out a selection bias due to an early switch to other treatments in patients that did not achieve a response with infliximab and were not included, as maintenance of a stable dose for at least 3 months was required for inclusion. On the other hand, the exclusion of patients with immunogenicity and the administration of infliximab doses adjusted by weight, and the presence of normal albumin and CRP values ​​suggests that patients treated with infliximab in our study had a low pharmacokinetic variability.

Eight patients treated with infliximab had concentrations above the recommended therapeutic range with no clinical or analytical differences with the in-range group other than gender, which we consider an incidental finding. Infliximab levels were negatively associated with serum concentrations of TNF-α, IL-12, and interferon-γ and positively with IL-10. These results are consistent with those previously observed by our group (Piñero et al., 2017; Zapater et al., 2019).

Higher IL-10 concentrations and lower IFN-γ concentrations were observed in patients with infliximab concentrations above the recommended therapeutic range and higher IL-26 in patients on intensified infliximab schedules. Increased IL-10 concentrations in the serum (Zapater et al., 2019), reduced production of interferon-γ at the cellular level in the intestinal mucosa (Agnholt and Kaltoft, 2001), and an increased production of IL-26 in the inflamed intestinal mucosa (Dambacher et al., 2009; Fujii et al., 2017) have been described in patients treated with infliximab, but our results show that this increase at the systemic level occurs according to drug concentrations and doses. The significance of this finding is yet to be determined.

Clinically, the evolution of the patients in the 6-month follow-up was similar in all patients, which is consistent with the observation that all patients had infliximab concentrations above the minimum concentrations associated with efficacy (Mitrev et al., 2017; Papamichael et al., 2019).

In case of adalimumab, the distribution of drug concentrations in the cohort of patients adjusted very well to that expected from the pharmacokinetic models. Up to 50% of the patients had serum drug concentrations below the recommended range. Other studies have described 30% of patients with subtherapeutic levels of adalimumab, although the concentration ranges may vary between studies (Carlsen et al., 2018; Reinhold et al., 2020) due to the present uncertainty regarding the consideration of “therapeutic concentration”.

In our study, body weight was the only clinical parameter that was significantly associated with trough concentrations of adalimumab. This is in agreement with previous studies that have associated the weight and the subcutaneous administration of adalimumab with the interindividual variability of adalimumab levels (Vande Casteele et al., 2019). Of special interest is the observation that this relationship is very strong in patients on intensified doses. Whether the administration of higher doses in obese patients with thicker subcutaneous fatty tissue alters the subcutaneous absorption of adalimumab is yet to be analyzed in specific drug absorption studies.

Similarly to infliximab, patients treated with adalimumab showed different changes in serum cytokines according to drug levels or doses. Patients who did not reach the adalimumab recommended levels had lower concentrations of IL-10 according to previous studies (Piñero et al., 2017; Zapater et al., 2019). Patients on intensified doses showed a significant increase in IL-12 levels, an important cytokine in the regulation of intestinal inflammation (Friedrich et al., 2019). It remains to be clarified whether the high levels of IL-12 are a consequence of poor disease control or a direct effect of high doses of adalimumab.

Our study has several limitations to consider. First, the risk of selection bias, arising from the decision to treat a particular patient with infliximab or adalimumab cannot be ruled out. That is why, the results of each drug have been presented separately. In addition, only one serum sample per patient was available, and there may have been changes in drug and cytokine levels at 6 months of follow-up. Secondly, classifying patients according to whether they are in a predefined range of concentrations may modify the results depending on the chosen ranges. Given this risk, we decided to choose the ranges established in the most recent consensus.

In conclusion, CD patients treated with infliximab and adalimumab exhibit different systemic concentrations of cytokines depending on the drug, dose intensification, and concentrations reached in the serum. These differences could be associated with clinical differences as shown by a higher number of hospitalization in patients treated with adalimumab who do not reach concentrations in the range (up to 50% of patients) or requiring intensified doses. These data support the need to consider the drugs and doses administered and the drug concentrations achieved in any future study that analyzes immune and clinical responses in CD patients.

## Data Availability

The original contributions presented in the study are included in the article/Supplementary Material; further inquiries can be directed to the corresponding author.
