# Supplementary figures and images for: Clinical and Immunological Factors Associated with Recommended Trough Levels of Adalimumab and Infliximab in Patients with Crohn’s Disease
Source: Front Pharmacol. 2022 Jan 3;12:795272. doi: 10.3389/fphar.2021.795272 (PMC8762261; doi:10.3389/fphar.2021.795272)

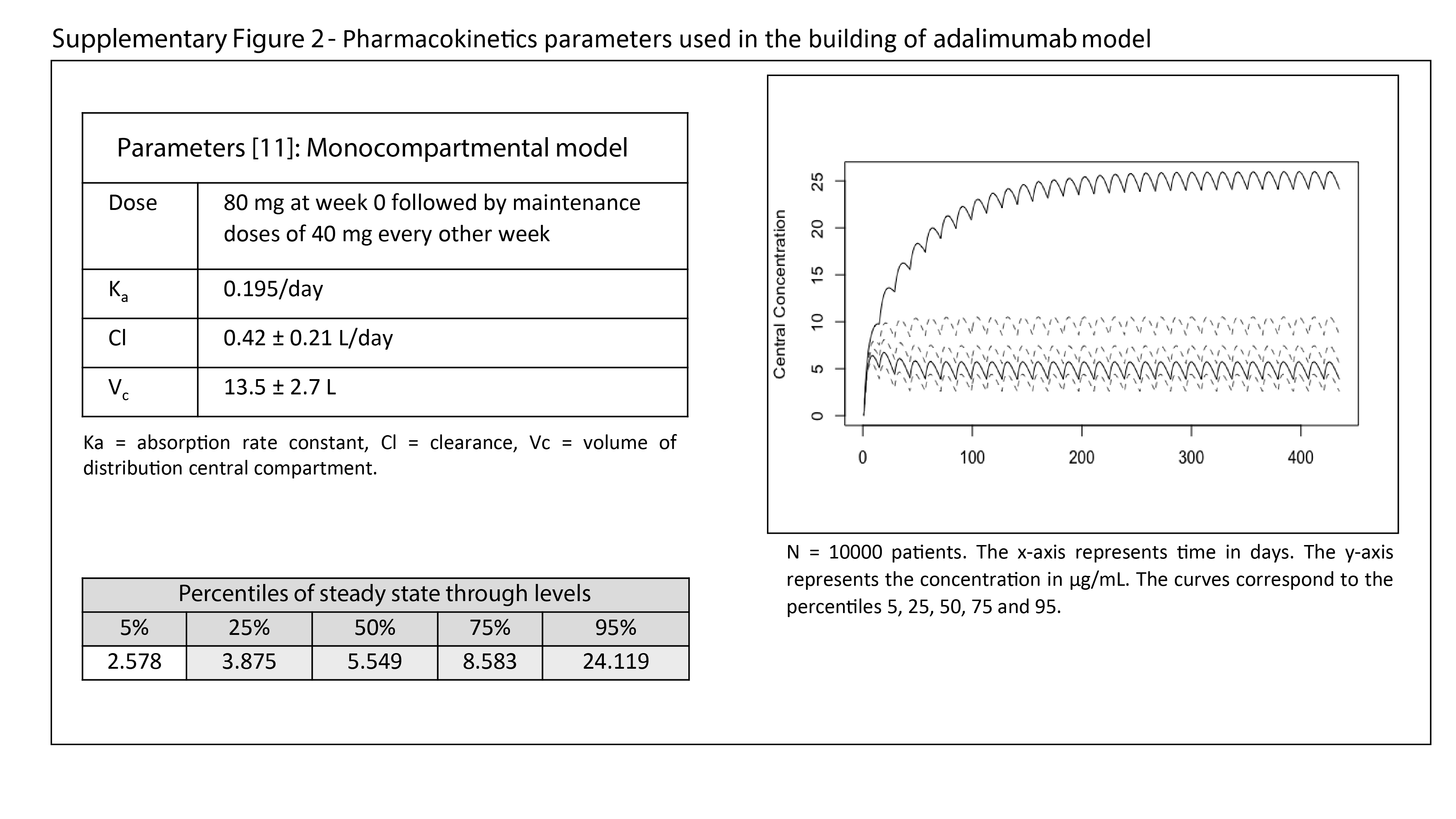

Supplement: Supplementary file 1 [file Image2.TIF]

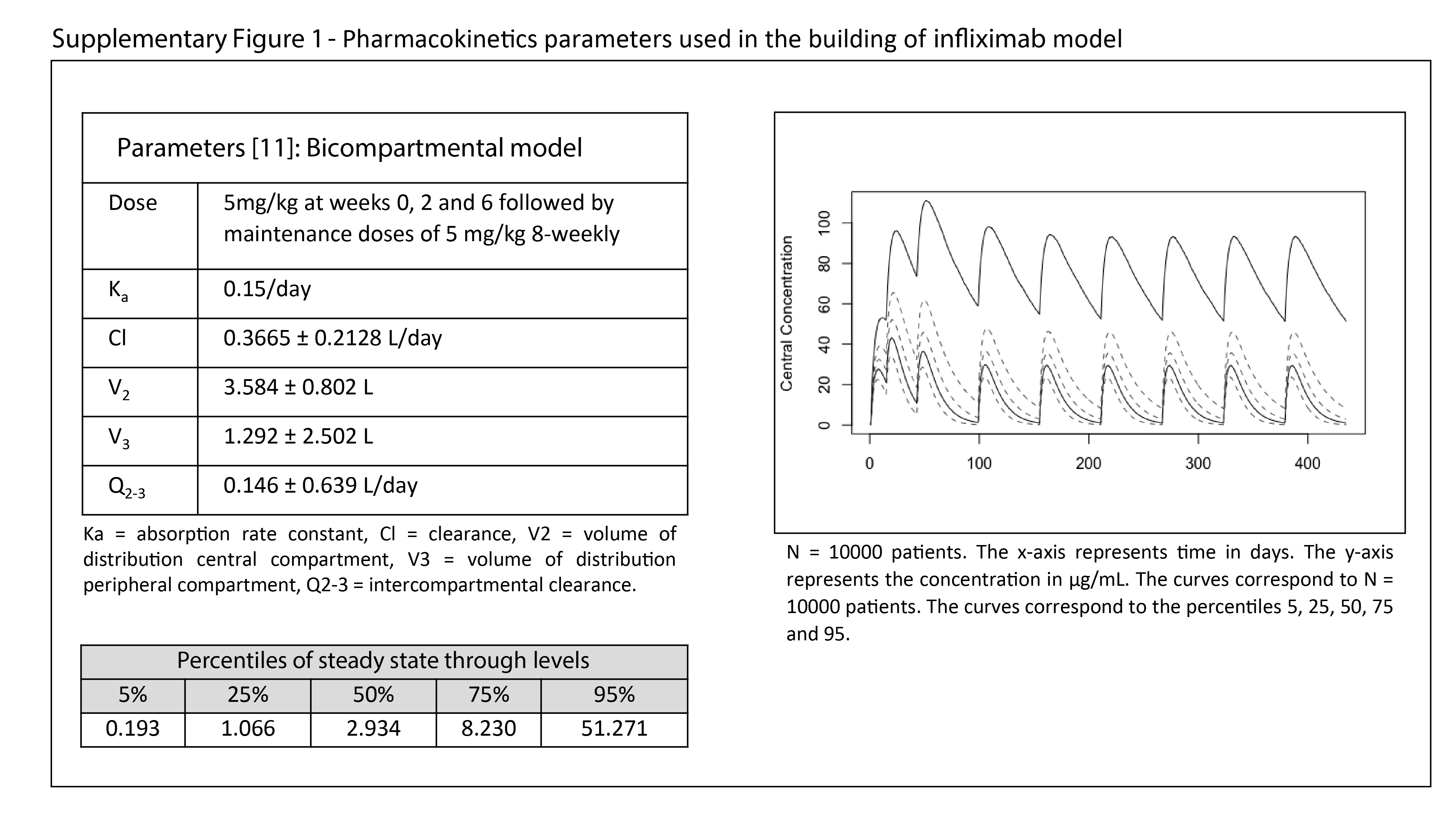

Supplement: Supplementary file 2 [file Image1.TIF]
