# Supplementary material for: Clinical and Immunological Factors Associated with Recommended Trough Levels of Adalimumab and Infliximab in Patients with Crohn’s Disease
Source: Front Pharmacol. 2022 Jan 3;12:795272. doi: 10.3389/fphar.2021.795272 (PMC8762261; doi:10.3389/fphar.2021.795272)
Supplement: Supplementary file 3 [file DataSheet1.docx]

**Supplementary table 1:** Characteristics of Crohn’s disease patients treated with infliximab according to dose and steady state trough levels range

|  | **3-8 µg/mL** | **Levels > 8 µg/mL** | **Intensified dose** |
| --- | --- | --- | --- |
| **N** | 47 | 7 | 8 |
| **Infliximab (µg/mL)** | 4.00 [3.30, 6.10] | 8.50 [8.20, 8.80] * | 4.80 [2.88, 5.35] |
| **Illness duration (months)** | 96.00 [48.00, 142.00] | 156.00 [78.00, 175.00] | 72.50 [45.00, 128.25] |
| **Age (yr)** | 36 ± 14 | 34 ± 14 | 38 ± 15 |
| **Female gender (%)** | 28 (59.6) | 0 (0.0) * | 4 (50.0) |
| **Weight (Kg)** | 74 ± 19 | 68 ± 21 | 73 ± 26 |
| **Current smokers yes (%)** | 16 (34.0) | 3 (42.9) | 4 (57.1) |
| **CRP** | 0.31 [0.10, 0.76] | 0.74 [0.15, 1.39] | 1.33 [0.23, 2.05] |
| **CDAI** | 89.00 [66.25, 159.75] | 105.00 [50.00, 171.00] | 144.00 [79.75, 304.50] |
| **Previous surgery (%)** | 9 (19.1) | 1 (14.3) | 3 (37.5) |
| **Calprotectin** | 64.00 [50.00, 215.00] | 110.00 [56.25, 266.25] | 285.00 [112.00, 537.50] |
| **Albumin (g/dL)** | 4.0 [3.7, 4.4] | 3.9 [3.8, 4.4] | 3.8 [2.9, 4.0] |
| **Hematocrit (%)** | 42.3 [40.4, 44.1] | 39.0 [36.6, 40.7] | 40.6 [37.4, 43.9] |
| **Leucocytes (/mm^3^)** | 6955 [5285, 8725] | 6610 [5810, 8600] | 6835 [6233, 7655] |
| **Montreal (age of onset), No. (%)** |  |  |  |
| **A1** | 7 (14.9) | 0 (0.0) | 1 (12.5) |
| **A2** | 32 (68.1) | 7 (100.0) | 5 (62.5) |
| **A3** | 8 (17.0) | 0 (0.0) | 2 (25.0) |
| **Montreal (location), No. (%)** |  |  |  |
| **L1** | 11 (23.4) | 4 (57.1) | 3 (37.5) |
| **L2** | 14 (29.8) | 1 (14.3) | 4 (50.0) |
| **L3** | 19 (40.4) | 1 (14.3) | 0 (0.0) |
| **L4** | 3 (6.4) | 1 (14.3) | 1 (12.5) |
| **Montreal (behavior), No. (%)** |  |  |  |
| **B1** | 30 (63.8) | 4 (57.1) | 6 (75.0) |
| **B2** | 7 (14.9) | 2 (28.6) | 1 (12.5) |
| **B3** | 10 (21.3) | 1 (14.3) | 1 (12.5) |
| **NSAID, No. (%)** | 2 (4.3) | 0 (0.0) | 1 (12.5) |
| **Azathioprine, No. (%)** | 19 (40.4) | 3 (42.9) | 1 (12.5) |
| **Steroids, No. (%)** | 6 (12.8) | 0 (0.0) | 0 (0.0) |

Numerical variables are shown as mean ± standard deviation or medians [interquartile range]. Categorical variables are described as frequency (percentage). * p < 0.05 vs. 3-8 µg/mL group. P values of differences between variables are calculated using the Mann-Whitney U test for continuous variables and the Fisher test for categorical variables.

**Supplementary table 2:** cytokine panel results in Crohn’s disease patients treated with infliximab according to steady state trough levels range

|  | **3-8 µg/mL** | **Levels > 8 µg/mL** | **Intensified dose** |
| --- | --- | --- | --- |
| **N** | 47 | 7 | 8 |
| **TNF-α** | 80.06 [52.12, 96.30] | 60.80 [32.58, 72.72] | 78.57 [74.90, 85.01] |
| **IL-26** | 20.34 [15.27, 33.06] | 20.75 [20.29, 21.50] | 91.80 [75.58, 109.45] * |
| **IL-10** | 26.15 [22.59, 37.98] | 84.00 [77.03, 84.80] * | 23.15 [22.27, 27.15] |
| **interferon-γ** | 405.58 [352.22, 526.56] | 312.90 [282.70, 350.36] * | 495.38 [338.40, 733.86] |
| **IL-12** | 558.90 [488.75, 740.75] | 558.80 [507.75, 573.25] | 535.78 [480.38, 761.58] |

Numerical variables are shown as median [interquartile range]. * p < 0.05 vs. 3-8 µg/mL group. P values of differences between variables are calculated using the Mann-Whitney U test.

**Supplementary table 3:** Characteristics of Crohn’s disease patients treated with adalimumab according to dose and steady state trough levels range

|  | **<5 µg/mL** | **Levels 5-12 µg/mL** | **Intensified dose** |
| --- | --- | --- | --- |
| **N** | 21 | 21 | 7 |
| **adalimumab (µg/mL)** | 3.70 [3.30, 4.50] | 6.50 [5.70, 6.70] * | 5.40 [3.20, 7.40] |
| **Illness duration (months)** | 96.00 [60.00, 228.00] | 120.00 [57.50, 228.00] | 56.00 [47.00, 76.50] |
| **Age (yr)** | 40 ± 11 | 41 ± 14 | 40 ± 14 |
| **Female gender (%)** | 12 (57.1) | 9 (42.9) | 4 (57.1) |
| **Weight (Kg)** | 76 ± 24 | 67 ± 13 | 70 ± 16 |
| **Current smokers yes (%)** | 8 (38.1) | 7 (33.3) | 2 (28.6) |
| **CRP** | 0.59 [0.16, 1.29] | 0.30 [0.16, 1.70] | 0.14 [0.11, 0.17] |
| **CDAI** | 86.00 [53.00, 123.50] | 89.00 [50.00, 232.00] | 63.00 [50.00, 127.00] |
| **Previous surgery (%)** | 8 (38.1) | 10 (47.6) | 0 (0.0) |
| **Calprotectin** | 55.00 [37.00, 88.00] | 85.00 [41.00, 215.00] | 55.50 [37.25, 75.25] |
| **Albumin (g/dL)** | 4.2 [3.9, 4.4] | 4.0 [3.4, 4.2] | 4.3 [3.5, 4.5] |
| **Hematocrit (%)** | 42.4 [38.9, 44.4] | 40.3 [38.6, 43.1] | 43.6 [38.2, 45.6] |
| **Leucocytes (/mm^3^)** | 8100 [7270, 11200] | 7705 [5140, 9295] | 7300 [5115, 9120] |
| **Montreal (age of onset), No. (%)** |  |  |  |
| **A1** | 2 (9.5) | 0 (0.0) | 0 (0.0) |
| **A2** | 18 (85.7) | 19 (90.5) | 6 (85.7) |
| **A3** | 1 (4.8) | 2 (9.5) | 1 (14.3) |
| **Montreal (location), No. (%)** |  |  |  |
| **L1** | 6 (28.6) | 9 (42.9) | 1 (14.3) |
| **L2** | 3 (14.3) | 4 (19.0) | 1 (14.3) |
| **L3** | 12 (57.1) | 6 (28.6) | 4 (57.1) |
| **L4** | 0 (0.0) | 2 (9.5) | 1 (14.3) |
| **Montreal (behavior), No. (%)** |  |  |  |
| **B1** | 12 (57.1) | 11 (52.4) | 2 (28.6) |
| **B2** | 3 (14.3) | 3 (14.3) | 2 (28.6) |
| **B3** | 6 (28.6) | 7 (33.3) | 3 (42.9) |
| **NSAID, No. (%)** | 0 (0.0) | 1 (4.8) | 0 (0.0) |
| **Azathioprine, No. (%)** | 1 (4.8) | 4 (19.0) | 2 (28.6) |
| **Steroids, No. (%)** | 4 (19.0) | 4 (19.0) | 1 (14.3) |

Numerical variables are shown as median [IQR]. Categorical variables are described as frequency (percentage). * p < 0.05 vs. < 5 µg/mL group. P values of differences between variables are calculated using the Mann-Whitney U test for continuous variables and the Fisher test for categorical variables.

**Supplementary table 4:** cytokine panel results in Crohn’s disease patients treated with adalimumab according to steady state trough levels range

|  | **<5 µg/mL** | **Levels 5-12 µg/mL** | **Intensified dose** |
| --- | --- | --- | --- |
| **N** | 21 | 21 | 8 |
| **TNF-α** | 82.25 [67.40, 98.83] | 70.40 [30.01, 88.90] | 95.78 [86.39, 96.03] |
| **IL-26** | 46.40 [17.05, 97.90] | 23.90 [16.42, 69.85] | 57.75 [35.03, 90.75] |
| **IL-10** | 26.32 [21.63, 33.21] * | 43.31 [35.32, 54.00] | 32.65 [18.88, 43.94] |
| **interferon-γ** | 355.66 [312.60, 658.80] | 371.42 [348.70, 570.22] | 350.40 [328.65, 718.06] |
| **IL-12** | 522.60 [476.49, 602.34] | 509.82 [473.78, 550.45] | 612.30 [570.20, 1353.72] * |

Numerical variables are showed as median [IQR]. * p < 0.05 vs. 5-12 µg/mL group. P values of differences between variables are calculated using the Mann-Whitney U test.
